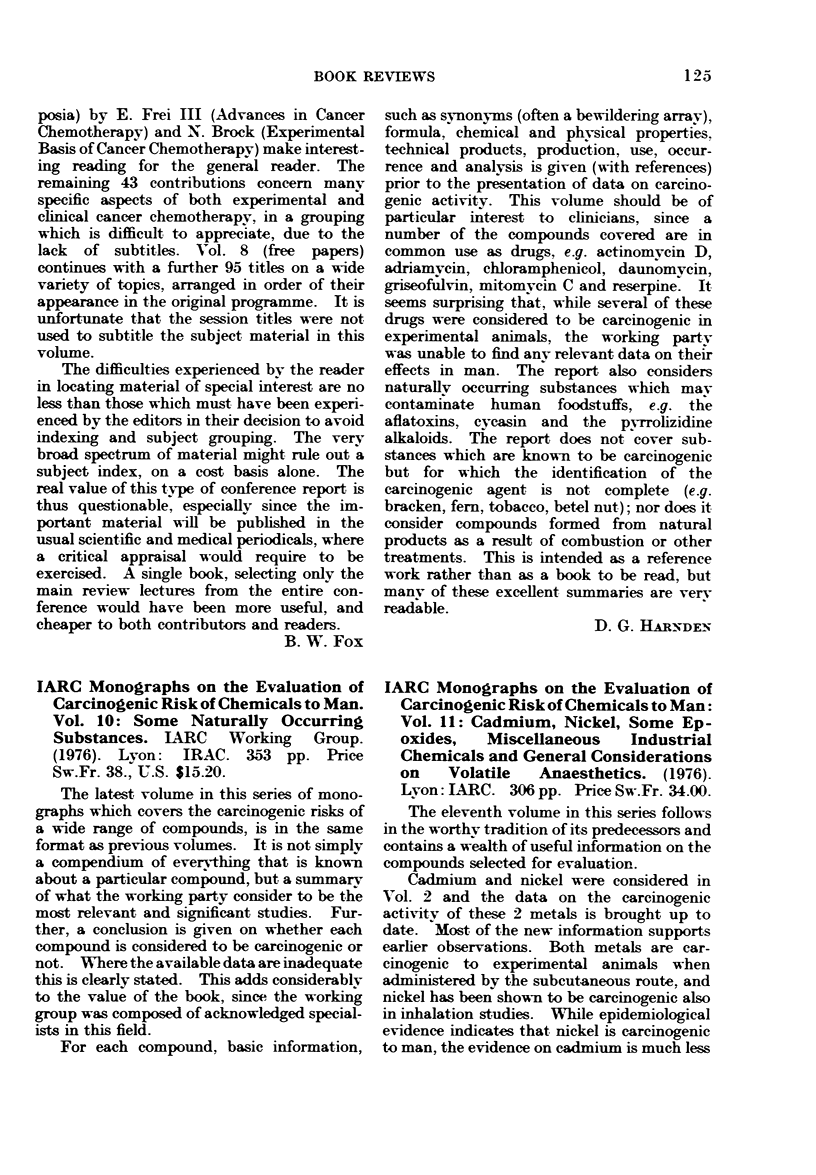# IARC Monographs on the Evaluation of Carcinogenic Risk of Chemicals to Man. Vol. 10: Some Naturally Occurring Substances

**Published:** 1977-01

**Authors:** D. G. Harnden


**IARC Monographs on the Evaluation of Carcinogenic Risk of Chemicals to Man. Vol. 10: Some Naturally Occurring Substances.** IARC Working Group. (1976). Lyon: IRAC. 353 pp. Price Sw.Fr. 38., U.S. $15.20.

The latest volume in this series of monographs which covers the carcinogenic risks of a wide range of compounds, is in the same format as previous volumes. It is not simply a compendium of everything that is known about a particular compound, but a summary of what the working party consider to be the most relevant and significant studies. Further, a conclusion is given on whether each compound is considered to be carcinogenic or not. Where the available data are inadequate this is clearly stated. This adds considerably to the value of the book, since the working group was composed of acknowledged specialists in this field.

For each compound, basic information, such as synonyms (often a bewildering array), formula, chemical and physical properties, technical products, production, use, occurrence and analysis is given (with references) prior to the presentation of data on carcinogenic activity. This volume should be of particular interest to clinicians, since a number of the compounds covered are in common use as drugs, *e.g.* actinomycin D, adriamycin, chloramphenicol, daunomycin, griseofulvin, mitomycin C and reserpine. It seems surprising that, while several of these drugs were considered to be carcinogenic in experimental animals, the working party was unable to find any relevant data on their effects in man. The report also considers naturally occurring substances which may contaminate human foodstuffs, *e.g.* the aflatoxins, cycasin and the pyrrolizidine alkaloids. The report does not cover substances which are known to be carcinogenic but for which the identification of the carcinogenic agent is not complete (*e.g.* bracken, fern, tobacco, betel nut); nor does it consider compounds formed from natural products as a result of combustion or other treatments. This is intended as a reference work rather than as a book to be read, but many of these excellent summaries are very readable.

D. G. Harnden